# Gender differences in scientific collaborations: Women are more egalitarian than men

**DOI:** 10.1371/journal.pone.0176791

**Published:** 2017-05-10

**Authors:** Eduardo B. Araújo, Nuno A. M. Araújo, André A. Moreira, Hans J. Herrmann, José S. Andrade

**Affiliations:** 1 Departamento de Física, Universidade Federal do Ceará, Campus do Pici 60451-970 Fortaleza, Ceará, Brazil; 2 Instituto Federal de Educação, Ciência e Tecnologia do Ceará, Campus Acaraú 62580-000 Acaraú, Ceará, Brazil; 3 Centro de Física Teórica e Computacional, Faculdade de Ciências, Universidade de Lisboa, Campo Grande, 1749-016 Lisboa, Portugal; 4 Departamento de Física, Faculdade de Ciências, Universidade de Lisboa, Campo Grande, 1749-016 Lisboa, Portugal; 5 Computational Physics for Engineering Materials, IfB, ETH Zurich, Wolfgang-Pauli-Strasse 27, CH-8093 Zurich, Switzerland; Northwestern University, UNITED STATES

## Abstract

By analyzing a unique dataset of more than 270,000 scientists, we discovered substantial gender differences in scientific collaborations. While men are more likely to collaborate with other men, women are more egalitarian. This is consistently observed over all fields and regardless of the number of collaborators a scientist has. The only exception is observed in the field of engineering, where this gender bias disappears with increasing number of collaborators. We also found that the distribution of the number of collaborators follows a truncated power law with a cut-off that is gender dependent and related to the gender differences in the number of published papers. Considering interdisciplinary research, our analysis shows that men and women behave similarly across fields, except in the case of natural sciences, where women with many collaborators are more likely to have collaborators from other fields.

## Introduction

The challenges faced by women in academia are considered to be responsible for their ubiquitous underrepresentation [1–4]. Signs of gender asymmetries are reported in several academic related activities such as hiring [5], grant funding [6], collaboration strategies [7], and even in the ordering of the list of authors in papers [8]. These studies are usually based on indirect analysis of scientific productivity [9–11] and the evaluation of their career strategy [4, 7, 12]. Here, we address the question of gender asymmetry from a different perspective. Many successful and high-impact research works result from the combination of skills, methods, and ideas of distinct team members. Thus the mechanisms of team building strongly affect the collaboration network structure and, consequently, its performance [13]. It is under this framework that we analyze a dataset with more than 270,000 scientists in Brazil and find that men are more likely to collaborate with other men than one would expect from the gender distribution across fields, while women are more egalitarian.

In order to apply for grants and fellowships at any career level, scientists in Brazil are required to register in the Lattes Platform [14]. This results in a very detailed public database, which includes all active scientists in Brazil and their full list of scientific publications. In contrast with other databases, in this platform, articles are uniquely identified by their DOI and possible ambiguities related to author names are practically solved [15, 16]. Besides, it also includes personal information such as gender, research field, and actual and previous academic positions. As a consequence, the application of network science methods [17–20] to the collaboration network can provide quantitative information for future discussions on the mechanisms responsible for the observed gender disparities [21].

The manuscript is organized as follows. In the next section, we present the results for the distribution of the number of collaborators, gender differences, and degree of research interdisciplinarity. Final conclusions and details about the methods are discussed afterwards.

## Materials and methods

For analyzing the collaboration patterns in Lattes Platform, the XHTML source code from circa 2.7 million curricula were extracted from the website [14] in June 2012. A parser was developed to extract information from the downloaded information.

When filling their curricula, scientists may choose up to three research fields. These are research topics organized in a hierarchical tree-like structure comprising eight major fields: *Agricultural Sciences* (AGR), *Applied Social Sciences* (SOC), *Biological Sciences* (BIO), *Exact* and *Earth Sciences* (EXA), *Humanities* (HUM), *Health Sciences* (HEA), *Engineering* (ENG) and *Linguistics and Arts* (LIN). Each of these have their own subfields. When assigning a specific field to a scientist, we considered the first displayed major field.

The procedure adopted to identify the collaborations is based on previous studies of the Lattes Platform [25]. A list, containing title, year of publication and number of authors of each paper published is created. More than 3.5 million papers are present in this list. The collaborations are identified by looking for duplicate records in the list. Due to typographical errors [26], an exact string matching would fail to identify collaborations. The Demearau-Levenshtein approximate string matching algorithm [27] is therefore used to define a distance between paper titles. Papers distant by less than 10% of the maximum distance, with the same number of authors and published in the same year are considered to be the same. Due to the extensive number of records, only papers published in the same year, with the same number of authors and starting with the same letter are compared. Following this procedure, more than 620 thousand collaborations were identified.

With a list of duplicate papers, a bipartite network *BN* is constructed containing two vertex classes, scientists (*R*) and papers (*P*). In this work, we analyze the projection of *BN* onto *R*, which we call TCN (Total Collaboration Network). Two scientists are said to collaborate if they are connected to a same paper in *BN*. The weight *w*_*ij*_ of their collaboration is defined as the number of papers in *BN* which both are connected to. We note that these networks are cumulative, with publications date spanning more than five decades. 11.5% of the scientists in TCN do not include field information on their curricula. The proportions of female scientists varies across fields [8] and the values for TCN are shown in Table 1.

**Table 1:** Number of scientists, fraction of the Total Collaboration Network (TCN) they represent and proportion of women for each of the eight major fields. The abbreviations are the same as in Table 2.

Field	Number of scientists (Fraction on TCN)	Female Proportion
AGR	31812 (11.6%)	44.4%
BIO	39767 (14.5%)	60.1%
HEA	67561 (24.6%)	59.8%
EXA	33310 (12.1%)	34.7%
HUM	26263 (9.55%)	65.1%
SOC	20806 (7.57%)	47.3%
ENG	18365 (6.68%)	27.2%
LIN	5202 (1.90%)	71.6%

Previous works on gender and collaboration [7, 12, 17, 23, 28] had information from a much smaller number of authors, usually much less than 10,000. Here, we have information concerning the productivity (as measured by article output) of 275,061 scientists with published papers on periodicals, 130,525 men (47.4%) and 144,440 women (52.5%). Only 96 scientists do not display the gender information on the curriculum. 90.4% belong to the giant component of the TCN.

## Results

**Fig 1:**
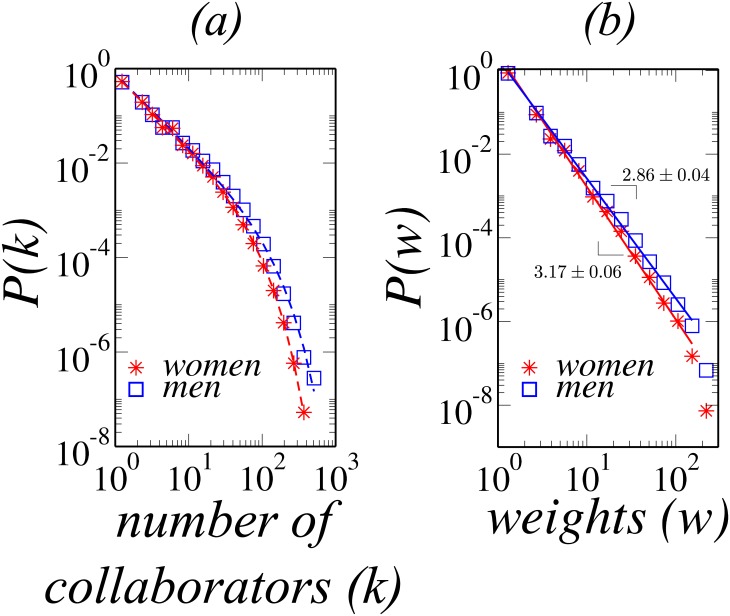
a) Distribution of the number of collaborators for men (blue squares) and women (red asterisks). The distributions are fitted with a truncated power law, *P*(*k*) = *Ak^−α^e^−k/β^*, plotted as dashed lines with colors corresponding to data points. The best fit is obtained for *α* = 1.53, and *β* = 85.4 and *β* = 49.5, for men and women, respectively, with r-squared 0.996 for men and 0.999 for women. b) Distribution of the number of recurrent collaborations between scientists (weights) for men (blue squares) and women (red asterisks). Solid lines are power-law fits, *P*(*w*) = *Bw^−λ^*, with colors corresponding to data points. For men, *λ* = 2.86 ± 0.04, while for women *λ* = 3.17 ± 0.06. With r-squared 0.997 for men and 0.996 for women.

The number of collaborators a scientist has is a cumulative quantity that depends on the entire scientific career. As shown in Fig 1a, the resulting distributions for men and women are consistent with a truncated power law, *P* = *Ak*^−*α*^*e*^−*k*/*β*^, with the same exponent *α* = 1.53 for both genders. However, the value of the parameter *β* for men is almost twice the one obtained for women, namely, 85.4 and 49.5, respectively. This difference reflects the tendency for men to have more collaborators than women.

In order to distinguish circumstantial from recurrent collaborations, we define the weight *w* of a collaboration between a pair of authors as the total number of papers co-authored by them. Fig 1b shows the distribution of weights for both genders. The least-squares fit of the data to a power law, *P*(*w*) = *Bw*^−*λ*^, gives *λ* = 3.17 ± 0.06 and *λ* = 2.86 ± 0.04, for men and women, respectively. This difference in *λ* might be related to the difference in the number of collaborators and papers. Table 2 summarizes the average number of collaborators and papers split by gender and research field. On average, men produce more papers and have more collaborators than women even in the fields where women are traditionally highly represented. This result confirms previous conclusions based on a dataset of 3,980 faculty members at U.S. universities [22]. An exception is the average number of collaborators in *Linguistics and Arts*, which is very similar for both genders.

**Table 2:** Mean number of collaborators and published papers for men and women for each of the eight major fields: *Agricultural Sciences* (AGR), *Applied Social Sciences* (SOC), *Biological Sciences* (BIO), *Exact and Earth Sciences* (EXA), *Humanities* (HUM), *Health Sciences* (HEA), *Engineering* (ENG) and *Linguistics and Arts* (LIN).

Field	Mean number of collaborators (women/men)	Mean number of papers (women/men)
AGR	9.20/13.6	9.45/17.5
BIO	10.9/14.9	10.0/17.7
HEA	7.65/11.2	9.18/17.7
EXA	7.90/9.84	9.49/15.6
HUM	3.16/3.31	7.54/11.4
SOC	2.85/3.57	6.62/10.5
ENG	6.02/6.50	8.08/11.0
LIN	2.06/2.04	8.25/11.2

**Fig 2:**
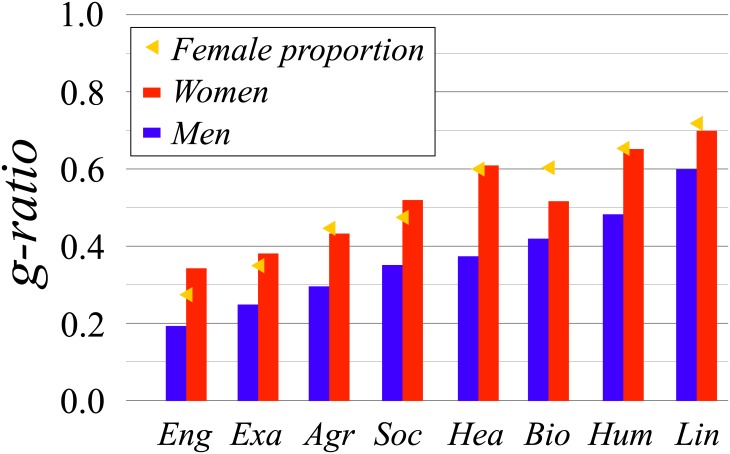
Mean values of the *g-ratio* across fields. Same abreviations for the fields as in Table 2. Blue (left) and red (right) bars represent values for men and women, respectively. Yellow triangles show the fraction of women working in the respective field. The error bars are smaller than 0.1% (see Table 3).

To evaluate homophily in the collaboration network, we define the gender ratio of a scientist *i*, *g-ratio*_*i*_, as

$$g\text{-}ratio_{i}=\frac{\sum_{j}^{\prime }w_{ij}}{\sum_{j}w_{ij}},$$ (1)

where the sum in the denominator is over all authors *j* with whom *i* as co-authored at least one publication, while the one in the numerator is only over those who are women, and *w*_*ij*_ is the weight of the collaboration between scientist *i* and *j*. Fig 2 depicts the average *g-ratio* for men and women across eight different fields. On average, women display a higher *g-ratio*, regardless of the field (see also Table 3). Men have relatively more collaborations with other men, indicating a tendency to a homophilic pattern. We also observe that the values of the *g-ratio* for women are always close to the fraction of women working in the respective field, while for men is significantly lower. Note that, the fraction of women working in the field would correspond to the expected value for the *g-ratio*, if collaborations were established at random. Previous results based on a rather small number of scientists suggested that women collaborate more with other women [12, 23], but we show that this is not the case for this much larger dataset.

**Table 3:** Average *g-ratio* for men and women for the eight major fields. Same abreviations for the fields as in Table 2.

Field	Female	Male
AGR	0.434 ± 0.002	0.297 ± 0.002
BIO	0.518 ± 0.002	0.421 ± 0.002
HEA	0.611 ± 0.002	0.375 ± 0.002
EXA	0.382 ± 0.003	0.250 ± 0.002
HUM	0.653 ± 0.003	0.484 ± 0.004
SOC	0.521 ± 0.004	0.353 ± 0.004
ENG	0.344 ± 0.004	0.194 ± 0.002
LIN	0.700 ± 0.007	0.601 ± 0.011

**Fig 3:**
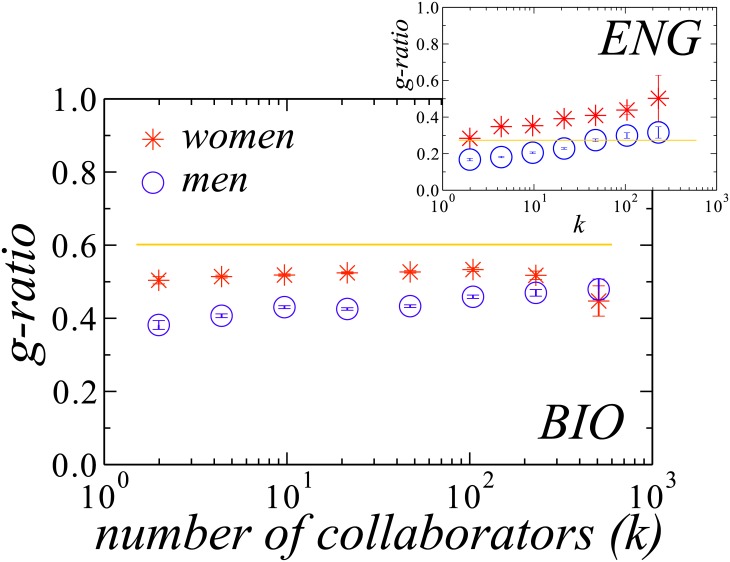
Relation between the *g-ratio* and the number of collaborators for *Biological Sciences* (BIO, main plot) and *Engineering* (ENG, inset) for women (red stars) and men (blue circles). Lines represent the fraction of women in the respective field. Men are more likely to collaborate with other men than with their female peers. For *Engineering*, the *g-ratio* is even above the fraction of women in the field. Error bars indicate the standard error for each bin.

The evidence of gender asymmetries raises the question of how it depends on the number of collaborators *k*, which is related to the career length. Except for *Engineering* (see inset in Fig 3), the *g-ratio* does not depend strongly on *k*. The values for women are always closer to the fraction of women in the respective field and the values for men are always consistently lower. This is exemplarily shown for *Biological Sciences* in Fig 3. For *Engineering*, collaboration with women grows continuously with *k* and even beyond the fraction of female scientists for men and women with more collaborators (see inset).

**Fig 4:**
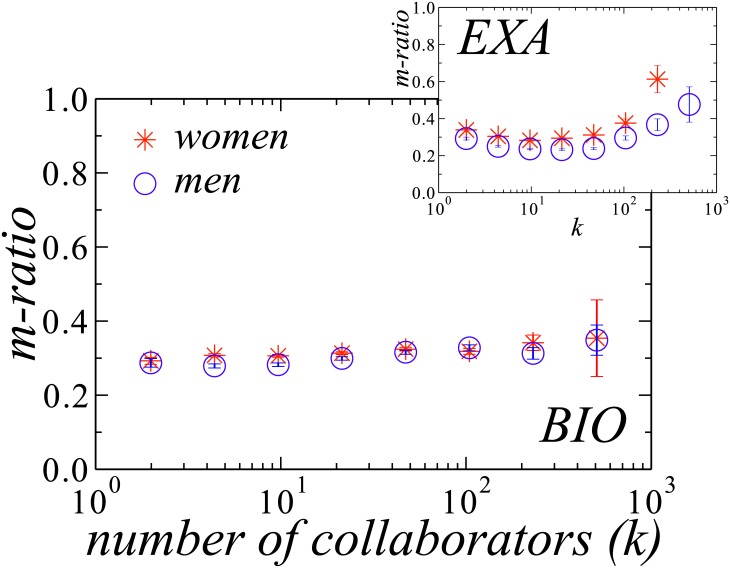
Dependence of the *m-ratio* on the number of collaborators in *Biological Sciences* (BIO, main plot) and *Exact and Earth Sciences* (EXA, inset) for women (red starts) and men (blue circles). Except for *Exact and Earth Sciences* (see inset), there is only a slight difference regarding multidisciplinary collaborations. Error bars indicate the standard error for each bin.

It has been reported that women are more involved in interdisciplinary research than their male peers [17, 24]. To evaluate this tendency in our dataset, we define the interdisciplinary ratio *m-ratio*_*i*_, as,

$$m\text{-}ratio_{i}=\frac{\sum_{j}^{\prime }w_{ij}}{\sum_{j}w_{ij}},$$ (2)

where the sum in the denominator is over all authors *j* with whom *i* as co-authored at least one publication, while the one in the numerator is only over those in a different field. The results are summarized in Table 4. We observe that women have more interdisciplinary collaborations than men for six fields, the exceptions being the fields of *Humanities* and *Linguistics and Arts*. The largest discrepancy is observed for *Exact and Earth Sciences*. Nonetheless, the differences are consistently smaller than the ones found for the *g-ratio*. We also calculated the *m-ratio* from Eq (2) considering in the numerator pairs of collaborators that have not declared any common field, as shown in Table 4. In this case, the values of the m-ratio are lower, but it is still clear that women have more interdisciplinary collaborations than men for six different fields. When analyzing the dependence on the number of collaborators, we observe the same tendency for men and women, as shown in Fig 4. However, for *Exact and Earth Sciences*, women with a larger number of collaborators (more than 100) are considerably more engaged in interdisciplinary research than men, with similar number of collaborators (see inset). This field dependence is very likely related to different collaborative norms in different fields.

**Table 4:** Average *m-ratio* for men and women for the eight major fields. The first two columns are obtained from Eq (2), where the sum in the numerator is over all co-authors with different major field, while, for the last two is over all co-authors without any field in common. Same abreviations for the fields as in Table 2.

	First field		All fields	
Field	Female	Male	Female	Male
AGR	0.225 ± 0.002	0.198 ± 0.002	0.065 ± 0.001	0.061 ± 0.001
BIO	0.309 ± 0.002	0.292 ± 0.002	0.090 ± 0.001	0.076 ± 0.001
HEA	0.187 ± 0.002	0.167 ± 0.002	0.064 ± 0.001	0.061 ± 0.001
EXA	0.305 ± 0.003	0.252 ± 0.002	0.090 ± 0.002	0.076 ± 0.001
HUM	0.290 ± 0.003	0.332 ± 0.004	0.082 ± 0.001	0.085 ± 0.002
SOC	0.282 ± 0.004	0.240 ± 0.004	0.090 ± 0.002	0.075 ± 0.002
ENG	0.331 ± 0.005	0.254 ± 0.003	0.086 ± 0.002	0.072 ± 0.001
LIN	0.274 ± 0.007	0.303 ± 0.011	0.081 ± 0.003	0.085 ± 0.005

## Conclusion

We have found gender differences regarding scientific collaborations in the Lattes Platform, a large dataset comprising more than 270,000 scientists. The number of collaborators and the weight of collaborations, measured in terms of the number of common publications, are both heavy tailed for men and women. Two metrics were introduced to investigate gender differences, namely, the *g-ratio*, that measures the fraction of collaborations with women, and the *m-ratio*, measuring the fraction of interdisciplinary collaborations.

With the *g-ratio*, we found that men collaborate more with other men than with women, and this happens systematically across different fields and regardless of their number of collaborators. The *m-ratio* analysis reveals that men and women have the same tendency to participate in interdisciplinary research, with women being slightly more engaged. For *Exact and Earth Sciences*, women with a larger number of collaborators are considerably more likely to work with scientists of a different field.

The path to gender balance in academia must involve not only government and institutional support, but also consciousness of the asymmetries in the current collaboration network. Our results are expected to provide quantitative support to future analyses and discussions. The specific causes for the homophilic pattern should also be investigated.
